# Developmental Comparison of Ceramide in Wild-Type and *Cln3*^Δ*ex7*/*8*^ Mouse Brains and Sera

**DOI:** 10.3389/fneur.2019.00128

**Published:** 2019-02-19

**Authors:** Sally El-Sitt, Jihane Soueid, Jamal Al Ali, Joelle Makoukji, Nadine J. Makhoul, Hayat Harati, Rose-Mary Boustany

**Affiliations:** ^1^Department of Biochemistry and Molecular Genetics, Faculty of Medicine, American University of Beirut, Beirut, Lebanon; ^2^Neuroscience Research Centre, Faculty of Medical Sciences, Lebanese University, Beirut, Lebanon

**Keywords:** CLN3 disease, ceramide, development, apoptosis, *Cln3*^Δ*ex7/8*^ mouse

## Abstract

CLN3 disease is a neurodevelopmental disease leading to early visual failure, motor decline, and death. CLN3 pathogenesis has been linked to dysregulation of ceramide, a key intracellular messenger impacting various biological functions. Ceramide is upregulated in brains of CLN3 patients and activates apoptosis. Ceramide levels over the lifespan of WT and *Cln3*^Δ*ex7/8*^ mice were measured using the DGK assay. Ceramide subspecies were determined by LC-MS. Ceramide synthesis enzymes and pre- and post-synaptic mRNA expression was measured in *Cln3*^Δ*ex7/8*^ and normal mouse brains. Neuronal cell death was established by PARP cleavage and Caspases 3/6/9 and cytochrome C mRNA expression in *Cln3*^Δ*ex7/8*^ and normal mouse brains. In WT mouse, a ceramide peak was noted at 3 weeks of age. The absence of this peak in *Cln3*^Δ*ex7/8*^ mice might be related to early disease pathogenesis. Increase of ceramide in *Cln3*^Δ*ex7/8*^ mouse brain at 24 weeks of age precedes neuronal apoptosis. The correlation between serum and brain ceramide in WT mice, and dysregulation of ceramide in serum and brain of *Cln3*^Δ*ex7/8*^ mice, and the significant increase in ceramide in *Cln3*^Δ*ex7/8*^ mouse brains and sera argue for use of easily accessible serum ceramide levels to track response to novel therapies in human CLN3 disease.

## Introduction

The neuronal ceroid lipofuscinoses (NCLs) are disorders that share abnormal accumulation of auto-fluorescent lipopigments in lysosomes of neurons and cells (1, 2). NCLs are pediatric neurodegenerative disorders and the leading cause of dementia in children (2). Infantile, late-infantile, and juvenile NCL correspond to CLN1/CLN2/CLN3 disease, respectively, and constitute the commonest forms of NCL. There are 13 recognized genetic variants at present.

CLN3 disease arises because of mutations in the *CLN3* gene. Its incidence is 1/25,000 births. Visual failure sets in at 4–6 years. This progresses to blindness by 10 years of age. Epilepsy, motor and cognitive decline, loss of speech, dystonia, psychiatric manifestations of anxiety and depression, and early death follow (3–5). Neurobiological hallmarks are elevated brain ceramide (6) determined prior to identification of the *CLN3* gene, neuronal and photoreceptor cell death, and accumulation of proteins, primarily subunit C of mitochondrial ATP synthase, in lysosomes of pyramidal neurons and other cells (7, 8).

*CLN3* encodes a 438 amino-acid anti-apoptotic protein (CLN3p) which impacts ceramide levels in cells at a molecular level, whereby overexpression lowers ceramide and knock-down increases ceramide levels *in vitro* (9, 10). CLN3p is implicated in anterograde transport of galactosylceramide (GalCer) from Golgi to lipid rafts (LRs) in the plasma membrane (PM) (7, 11). *CLN3*-defective cells lack GalCer in LRs/PM. The hypothesis is that an increase in ceramide, the building block for GalCer, in CLN3 brain (6) and CLN3-deficient cells (9, 10) is an attempt to compensate for the GalCer deficit in LRs (11). GalCer comprises 12% of myelin (12). CLN3p is synthesized in the lumen of the endoplasmic reticulum (ER) prior to transport to its final destination in plasma membrane and lipid rafts (13). A ~1 kb deletion eliminating exons 7/8 of *CLN3* gene is the commonest human mutation. The *Cln3*^Δ*ex7/8*^ knock-in mouse model (14) manifests biochemical, neuropathological, and behavioral changes consistent with human CLN3 disease (14).

Sphingolipids are important for membrane structure and regulation of cell function. Sphingolipid *de novo* synthesis occurs in ER (15). Serine/palmitate condense to 3-ketodihydrosphingosine mediated by serine palmitoyl transferase (SPTLC1). Rapid reduction of 3-ketodihydrosphingosine to dihydrosphingosine by 3-ketosphinganine reductase (KDSR) follows. Then, dihydrosphingosine is acylated by dihydroceramide synthases (CerS1–6) to dihydroceramide which is desaturated by dihydroceramide desaturase (DEGS) to ceramide (15).

Ceramide is a key modulator of sphingolipid metabolism and is the building block for complex sphingolipids and glycosphingolipids (16). Its structure involves long-chain bases attached to acyl chains via amide bonds. Length, hydroxylation, and saturation of the sphingoid base and fatty acids characterize ceramide species (17). Ceramide affects growth, differentiation, apoptosis, and oncogenesis and impacts subcellular and biochemical targets inducing cell cycle arrest and apoptosis (18–20). Apoptosis marks pathogenesis of neurodegeneration in NCL (6, 21, 22), amyotrophic lateral sclerosis, Alzheimer's, Huntington's, Parkinson's disease and other disorders (23, 24).

It was previously determined that CLN3 disease pathogenesis involves dysregulation of ceramide, which accumulates in brains of patients (6) and cells (9, 10), and it has been proven to activate the apoptosis pathway upstream of caspases in cells (21). Supplementing *CLN3*-defective cells with *CLN3*-DNA or exogenous GalCer abrogates apoptosis and restores GalCer levels to LRs, preventing elevation of ceramide levels (11).

Elevation of ceramide in sera of patients with CLN3 disease has also been established (El-Sitt et al., p. 74, NCL 2018 London Programme Abstract). Here, ceramide levels in wild-type (WT) and *Cln3*^Δ*ex7/8*^ sera and brains at different ages are established, for the first time, to gain insight into normal/abnormal temporal changes in ceramide in WT and *Cln3*^Δ*ex7/8*^ mice during development. The mRNA expression of ceramide metabolism enzymes and that of presynaptic/postsynaptic proteins of normal synaptogenesis in whole mouse brain are documented, as well as mouse brain cell death following peak ceramide levels. This will aid in elucidating the pathobiology of CLN3 disease and provide evidence for the potential use of ceramide as a serum marker for tracking CLN3 disease in response to therapies in humans.

## Materials and Methods

### Animals

The study was carried out at the Animal Care Facility of the American University of Beirut (AUB) and all animal experiments were performed in compliance with the AUB Institutional Animal Care and Use Committee (IACUC) guidelines (reference number: 17-03-RN407). Homozygous *Cln3*^Δ*ex7/8*^ mice, bred on a C57BL/6J background, were purchased from the Jackson laboratories (Bar Harbor, ME, US) in addition to WT C57BL/6J mice (reference number: 17-03-RN407). All animals are housed in a controlled environment with a temperature of 22–24°C, humidity of 60% and a 12 h light-dark cycle.

### Measurement of Ceramide Levels by DGK Assay

Brain Tissue Homogenization: mouse brains are suspended in 2 ml of homogenization medium (Tris 1 M, NaCl 1M, EDTA 100 mM, DTT 100 mM) with protease inhibitors (Sigma-Aldrich, MO, USA), disrupted by Dounce homogenization, and stored at −80° for 24 h.

Lipid extraction (Brain): 1,400 μl of distilled water are added to the homogenate followed by 2 ml chloroform and 2 ml of distilled water prior to centrifugation for 10 min at 1,000 × g at 4°C. The lower phase is lyophilized. Lipids are re-suspended in 1,000 μl of chloroform.

Lipid extraction (Serum): 3 volumes of 100% Methanol containing 30 mM ammonium acetate are added to the homogenate followed by 4 volumes of chloroform prior to centrifugation for 10 min at 1,000 × g at 4°C. The lower phase is lyophilized. Lipids are re-suspended in 1,000 μl of chloroform.

Ceramide Assay (Diacyl glycerol kinase (DGK) assay): DGK assay is a well-established method for ceramide measurement, and has been well-established in our laboratory (10, 11, 25), and is used worldwide. Ceramide standards (Non-hydroxy Fatty Acid Ceramide from Sigma-Aldrich, MO, USA) and samples are dried under vacuum. β-octylglucoside (BOG) (Sigma-Aldrich, MO, USA) and 1,2-dioleoyl-sn-glycero-3-phospho-(1′-rac-glycerol) (DOPG) (Sigma-Aldrich, MO, USA) are added as micelles mixture to samples/standards and sonicated for 30 min. Reaction mixtures are added to the ATP mix containing 1.3 μCi ATP, [γ-^32^P] (IZOTOP, Hungary) per sample and incubated at room temperature (RT) for 45 min. The reaction is stopped using methanol/chloroform/distilled water prior to lipid extraction. Lower phase is dried under vacuum, re-suspended in chloroform/methanol (9:1) and run on a TLC plate (SILICYCLE®, Canada) using chloroform/acetone/methanol/acetic acid/water as running buffer (50:20:15:10:5). Plates are dried, x-ray film overlaid and kept at −80°C overnight to visualize ceramide bands. Lanes of the TLC plate are scraped into scintillation vials, and counts/minute detected using a liquid scintillation counter. Results are expressed as pmols of ceramide/nmol of total phospholipids.

Phosphate determination: After lyophilization, 150 μL of 70% perchloric acid (Fluka®, Germany) is added to disodium hydrogen phosphate (Na_2_HPO_4_) standards (MERCK, NJ, USA), tubes capped with methanol-soaked glass balls, placed at 180°C for 1 h, cooled at RT and distilled water/2.5% ammonium molybdate (Honeywell Riedel-de Haën, Germany)/10% ascorbic acid (Biochemical) added. Mixtures were then incubated for 15 min at 50°C, and concentration determined at 820 nm by spectrophotometer. Ceramide concentration (pmol) is normalized to phosphate concentration (μg).

### Measurement of Ceramide Subspecies by LC/MS

Sphingolipids are analyzed from 20 mg mouse brain tissue by liquid chromatography/mass spectrometry (LC/MS) as previously described (26). The liquid chromatography system is a U3000 from Thermo Fisher Scientific (MA, USA) and the mass spectrometer is an Exactive high-resolution system (Thermo Fisher Scientific, MA, USA) equipped with an electrospray ionization source. For the analyses, 5 μL of each sample are injected. LC/MS analysis is performed at Metatoul: platform of metabolomics and fluxomics of the Genopole de Toulouse (France). Ceramide species (pmol) are normalized to total protein content (μg) as evaluated by Bradford assay (BioRad, CA, USA).

### RNA Isolation

Total RNA is isolated from brain tissue using the mirVana^TM^ miRNA Isolation Kit (Thermo Fisher Scientific, MA, USA) that allows isolation of total RNA with excellent yields. Experiments are performed according to manufacturer protocols followed by RNA cleanup using RNeasy® Plus Mini Kit (Qiagen, Germany). RNA is stored at −80°C. RNA concentrations and quality is assessed by analyzing A_260_/A_280_ and A_260_/A_230_ ratios with a ND-1,000 spectrometer (Nanodrop Technologies LLC, DE, USA).

### Quantitative Real-Time PCR

Total RNA is reverse transcribed using RevertAid Reverse Transcriptase (Thermo Fisher Scientific, MA, USA) with 100–1,000 ng of input RNA and random primers (Thermo Fisher Scientific, MA, USA). Quantitative real-time PCR reactions (qRT-PCR) are performed in 384-well plates using specific primers (TIB MOLBIOL, Germany) (Supplementary Table S1) and the iQTM SYBR® Green Supermix (BioRad, CA, USA) as a fluorescent detection dye in a final volume of 10 μl [CFX96TM Real-Time PCR (BioRad, CA, USA)]. Each reaction is performed in triplicate. All results are normalized to β*-actin* or *Gapdh* mRNA levels and calculated using the 2^−Δ*Ct*^ method. To characterize generated amplicons and to control contamination by unspecific by-products, melt curve analysis is applied.

### PARP Cleavage

Immunoblotting is performed by Western blot according to standard procedures using ECL detection. Tissue samples are run on SDS–PAGE (4–20% acrylamide) in a Tris-glycine running buffer system and transferred onto a PVDF membrane with a Tris-glycine buffer system using a wet transfer unit (Bio-Rad) at 30 volts overnight. The blots are probed with the monoclonal rabbit anti PARP primary antibody (Cell Signaling Technology #9532, 1/1000). Twenty microgram of protein are loaded per band. GAPDH (monoclonal #encor-mca1d4, 1/2000) are used as a protein loading evenness control. The blots are washed with TBST and exposed to HRP coupled secondary antibodies and results assessed using a Chemidoc machine (BioRad, CA, USA). The molecular weights of intact proteins are established by running against all blue molecular markers (#161-0373/ BioRad, CA, USA).

### Statistical Analysis

Statistical analysis is conducted using GraphPad Prism 6 software (GraphPad software, CA, USA). Continuous data is expressed as mean ± SEM. Statistical analysis is performed using two-tailed Student's *t-*test and one-way analysis of variance (ANOVA) followed by Tukey *post hoc* test for multiple group comparisons. Values of *p* < 0.05 are considered statistically significant.

## Results

### Ceramide Levels in CLN3 Disease

**Figure 1:**
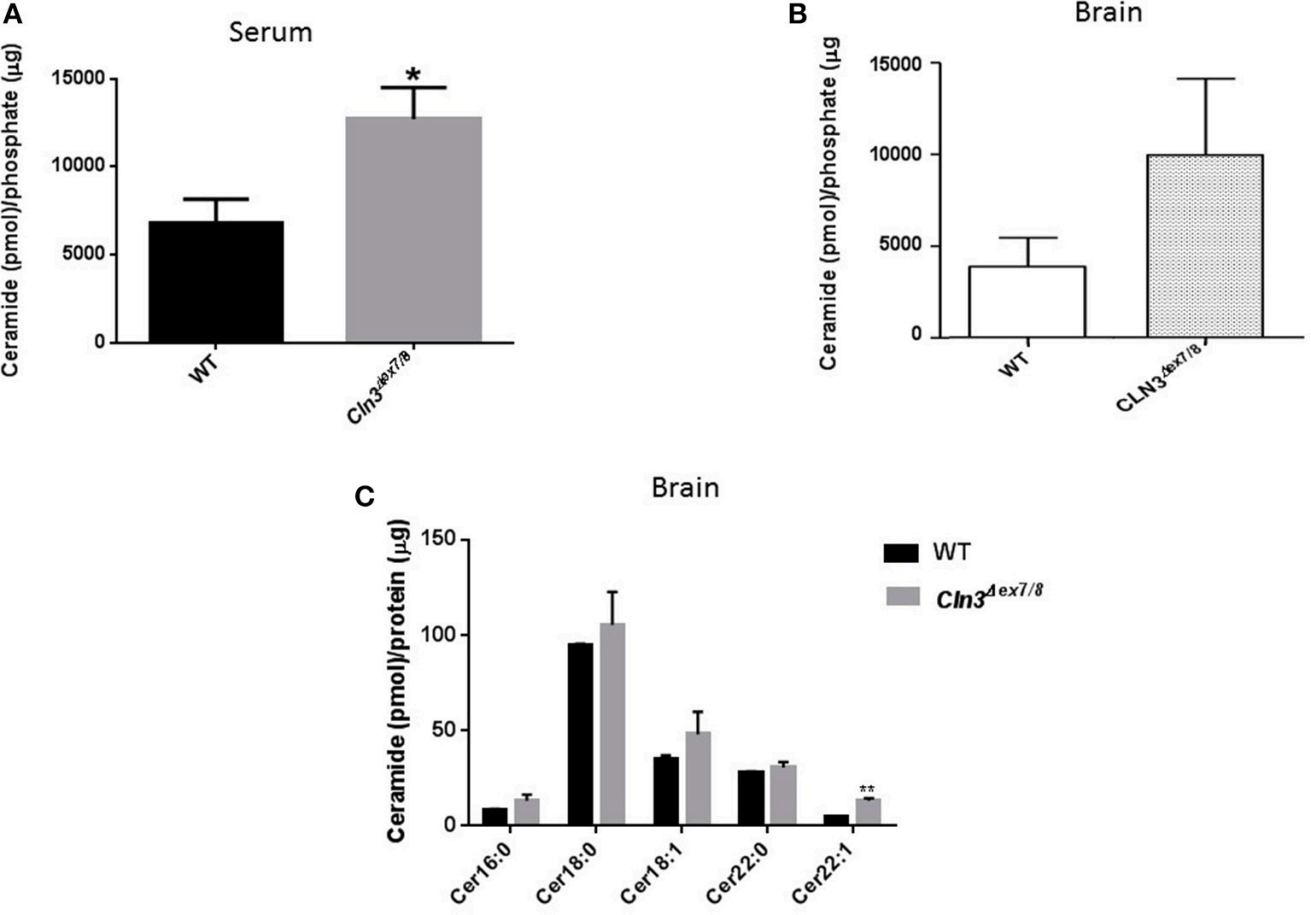
**(A)** Serum ceramide levels by DGK assay in age-matched WT and *Cln3*^Δ*ex7/8*^ mice (16, 24, and 48 weeks). Serum ceramide levels in *Cln3*^Δ*ex7/8*^ mice (*n* = 12) were significantly higher compared to age-matched WT mice (*n* = 7) (^*^P < 0.05). **(B)** Brain ceramide levels were determined by DGK assay in 17 weeks-old age-matched WT and *Cln3*^Δ*ex7/8*^ mice (*n* = 4 in each group). Brain ceramide levels in *Cln3*^Δ*ex7/8*^ mice were higher compared to age-matched WT mice. **(C)** Ceramide subspecies were characterized by LC-MS in 44 weeks-old age-matched WT and *Cln3*^Δ*ex7/8*^ mice (*n* = 4 in each group). Cer16, Cer18, Cer18:1, Cer22, and Cer22:1 were the major species identified in WT mouse brain, with Cer18 being the predominant ceramide fatty acid. In age-matched *Cln3*^Δ*ex*7/8^ mice, corresponding ceramide fatty acids had higher levels, with the difference statistically significant for Cer22:1 (^**^P < 0.01).

Sera and brains from WT and *Cln3*^Δ*ex7/8*^ mice were collected, and ceramide levels analyzed by DGK assay. Mean ceramide serum levels in *Cln3*^Δ*ex7/8*^ mice are significantly higher compared to mean serum levels in age-matched WT mice (Figure 1A). Mean ceramide brain levels in *Cln3*^Δ*ex7/8*^ mice are also higher compared to mean levels in age-matched WT mice (Figure 1B). This increase in ceramide has been documented in brains of CLN3 patients (6). Also, we showed that ceramide levels in serum of CLN3 patients is significantly higher compared to ceramide serum level in normal controls (El-Sitt et al., p. 74, NCL 2018 London Programme Abstract). Nowadays, there is considerable interest in quantifying levels of these complex lipids in physiologic and pathophysiologic states. Brain mouse tissues (WT and *Cln3*^Δ*ex*7/8^) were harvested prior to analysis by LC-MS for ceramide (Cer) species. Of 31 different ceramide and α-hydroxyl ceramide subspecies that were monitored, Cer16, Cer18, Cer18:1, Cer22, and Cer22:1 were the major species identified in WT mouse brain (Figure 1C). N-stearoyl (Cer18) ceramide was the predominant fatty acid in WT mouse brain. In age-matched *Cln3*^Δ*ex*7/8^ mice, these ceramide fatty acid levels were higher, and the difference was statistically significant for Cer22:1 (Figure 1C).

**Figure 2:**
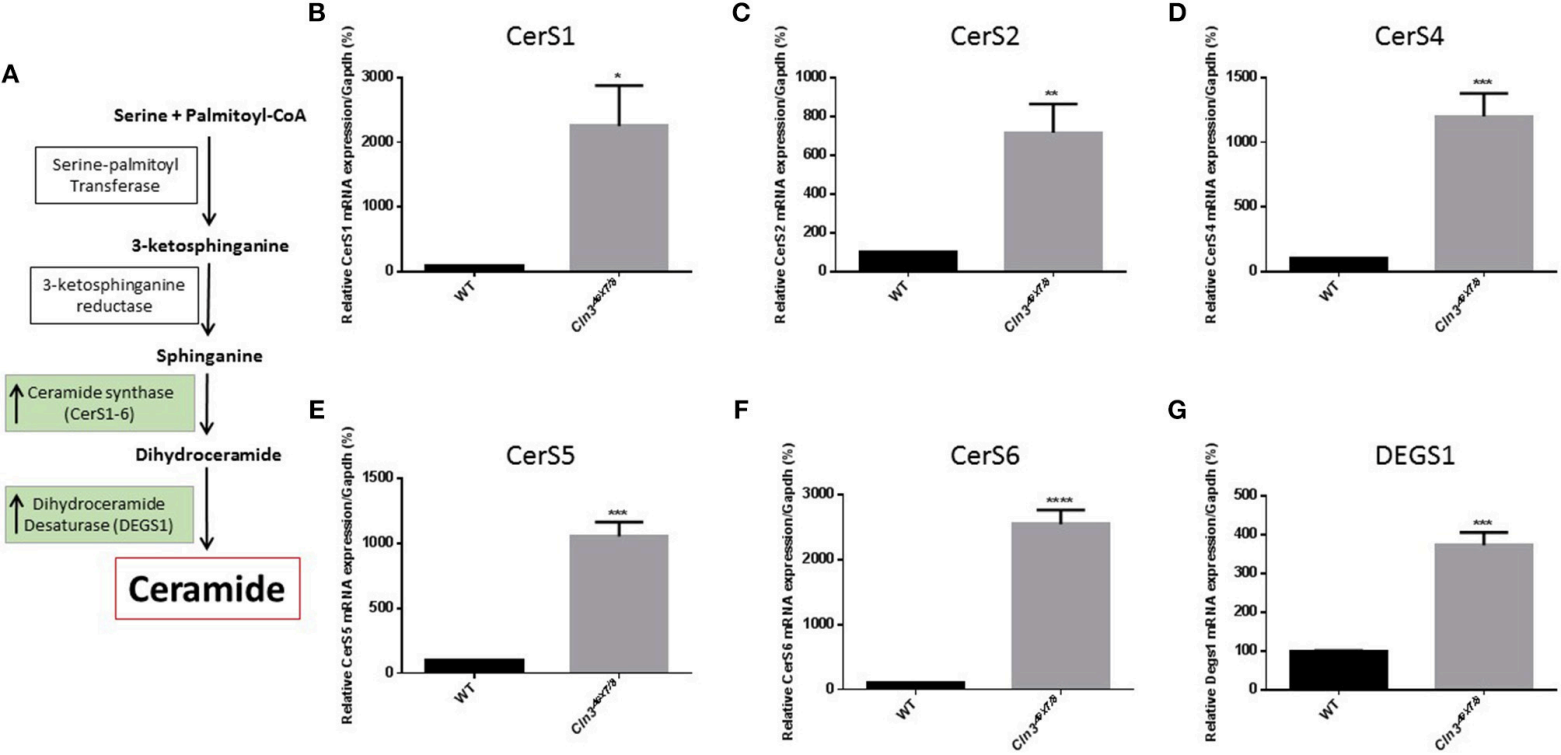
**(A)** *De novo* synthesis pathway for generation of ceramide in ER. Relative expression of *de novo* ceramide synthesis enzymes: CERS1 **(B)** (^*^P < 0.05), CERS2 **(C)** (^**^P < 0.01), CERS4 **(D)** (^***^P < 0.001), CERS5 **(E)** (^***^P < 0.001), CERS6 **(F)** (^****^P < 0.0001), and DEGS1 **(G)** (^***^P < 0.001) in WT and *Cln3*^Δ*ex7/8*^ mouse brain at 44 weeks of age (*n* = 4 in each group). Enzymes are substantially upregulated in *Cln3*^Δ*ex7/8*^ whole mouse brain compared to 44 weeks-old age-matched WT mice.

Differences in ceramide fatty acid chain length are probably due to variable expression levels and activity of ceramide synthases in WT vs. *Cln3*^Δ*ex7/8*^ mice. Whole mouse brain expresses at least three ceramide synthases (CerS1, 2, and 6). In humans, ceramide synthase 1 (CerS1) is the most specific for brain and mainly produces Cer18-ceramides (27). Ceramide is derived from several pathways operating in different cellular compartments. *De novo* synthesis is one of the major pathways that generates ceramide from serine and palmitoyl CoA in the endoplasmic reticulum (ER) (Figure 2A). WT and *Cln3*^Δ*ex7/8*^ mouse brain mRNA levels of *de novo* ceramide synthesis enzymes DEGS1 and ceramide synthases 1–6 (CerS1–6) were determined using qRT-PCR. Enzymes involved in *de novo* synthesis of ceramide (DEGS1, CerS1–6) are substantially upregulated in *Cln3*^Δ*ex7/8*^ whole mouse brain compared to age-matched WT mice (Figures 2B–G). This is in line with the increase of total ceramide levels in *Cln3*^Δ*ex7/8*^ mouse brain compared to WT mice.

### Ceramide Levels Over the Lifespan of WT and *Cln3^Δ**ex*7/8*^* Mice

**Figure 3:**
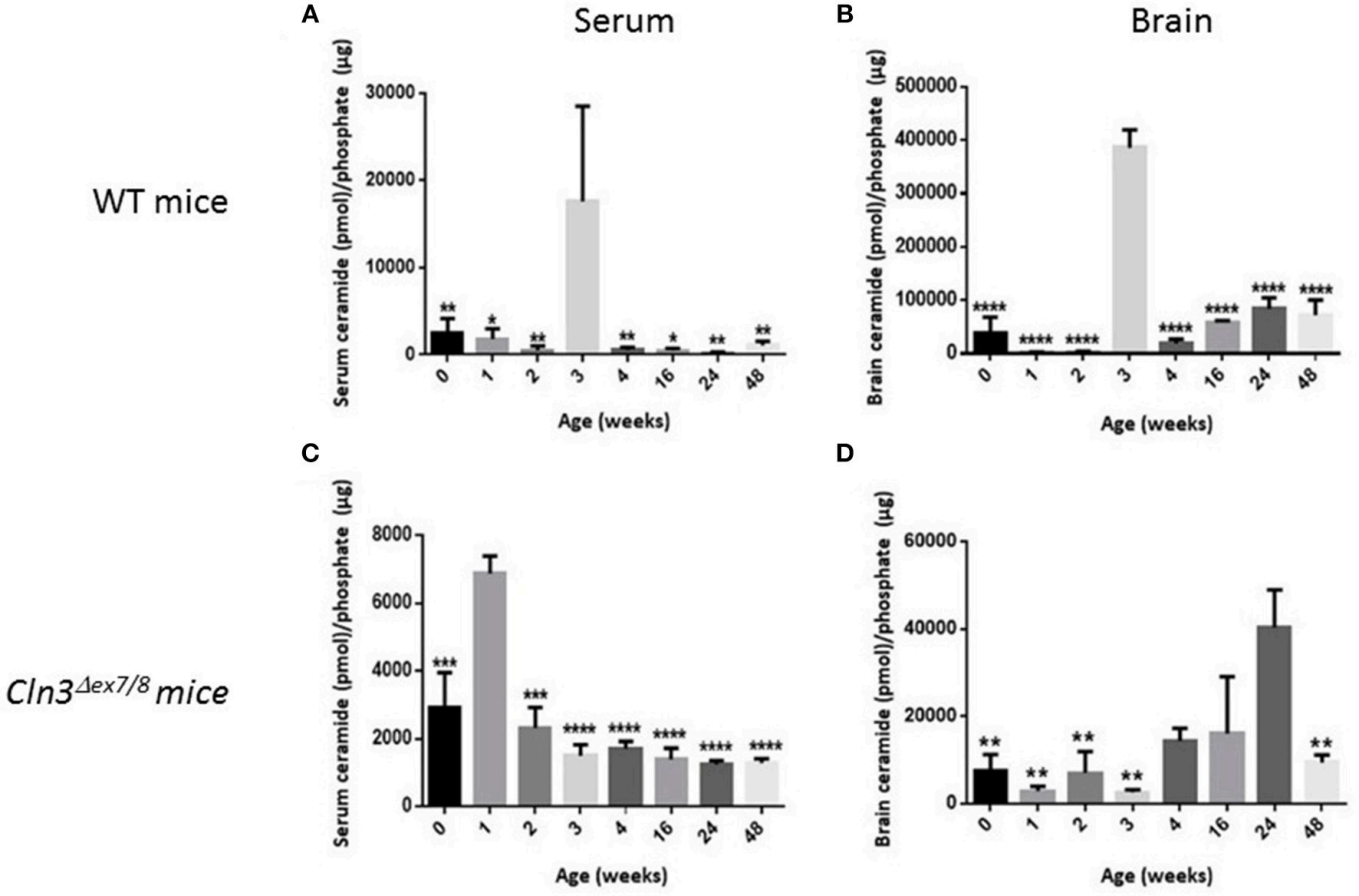
Ceramide levels determined by DGK assay in **(A)** WT mouse sera at different ages in weeks (at 0w: *n* = 6; 1w: *n* = 3; 2w: *n* = 3; 3w: *n* = 3; 4w: *n* = 6; 16w: *n* = 3; 24w: *n* = 3; 48w: *n* = 3) (^*^P < 0.05, ^**^P < 0.01). **(B)** WT mouse brain (at 0w: *n* = 5; 1w: *n* = 3; 2w: *n* = 3; 3w: *n* = 3; 4w: *n* = 6; 16w: *n* = 3; 24w: *n* = 3; 48w: *n* = 3) (^****^P < 0.0001). **(C)** *Cln3*^Δ*ex7/8*^ mouse sera (at 0w: *n* = 4; 1w: *n* = 3; 2w: *n* = 4; 3w: *n* = 3; 4w: *n* = 4; 16w: *n* = 4; 24w: *n* = 4; 48w: *n* = 4) (^***^P < 0.001, ^****^P < 0.0001). **(D)** *Cln3*^Δ*ex7/8*^ mouse brain (at 0w: *n* = 4; 1w: *n* = 4; 2w: *n* = 3; 3w: *n* = 4; 4w: *n* = 3; 16w: *n* = 3; 24w: *n* = 4; 48w: *n* = 4) (^**^P < 0.01). In WT mice **(A,B)**, brain ceramide levels correlate with serum ceramide levels. Ceramide levels in sera and brain peak at 3 weeks of age. In *Cln3*^Δ*ex7/8*^ mice **(C,D)**, ceramide brain levels did not temporally correlate with serum levels. In serum, ceramide level peaks significantly at 1 week of age, whereas, in brain, ceramide level peaks significantly at 24 weeks. In WT mice, statistical significance is computed relative to the peak at week 3 in **(A,B)**. In *Cln3*^Δ*ex7/8*^ mice, statistical significance is computed relative to week 1 peak in **(C)**, and week 24 peak in **(D)**.

Sera and brains were collected from WT mice at 0, 1, 2, 3, 4, 16, 24, and 48 weeks of age, and ceramide levels were analyzed by DGK assay. In normal WT mice, serum ceramide levels (Figure 3A) correlate positively with brain ceramide levels (Figure 3B). There is a significant ceramide peak at 3 weeks of age in serum and brain of WT mice, while ceramide levels remain relatively low and unchanged at 0, 1, 2, 4, 16, 24, and 48 weeks of age. Determining normative values of ceramide in WT mice enables benchmarking serum and brain ceramide levels in *Cln3*^Δ*ex7/8*^ mice. Sera and brains were collected from age-matched *Cln3*^Δ*ex7/8*^ mice. Ceramide serum levels do not correlate with brain levels in *Cln3*^Δ*ex7/8*^ mice. A significant peak in *Cln3*^Δ*ex7/8*^ mouse sera was established at 1 week of age (Figure 3C). A ceramide peak in *Cln3*^Δ*ex7/8*^ mouse brain is established at 24 weeks of age (Figure 3D). Relatively low and unchanged ceramide levels were detected at all other ages determined in both sera and brains.

### Expression Analysis of *de novo* Ceramide Synthesis Enzymes

**Figure 4:**
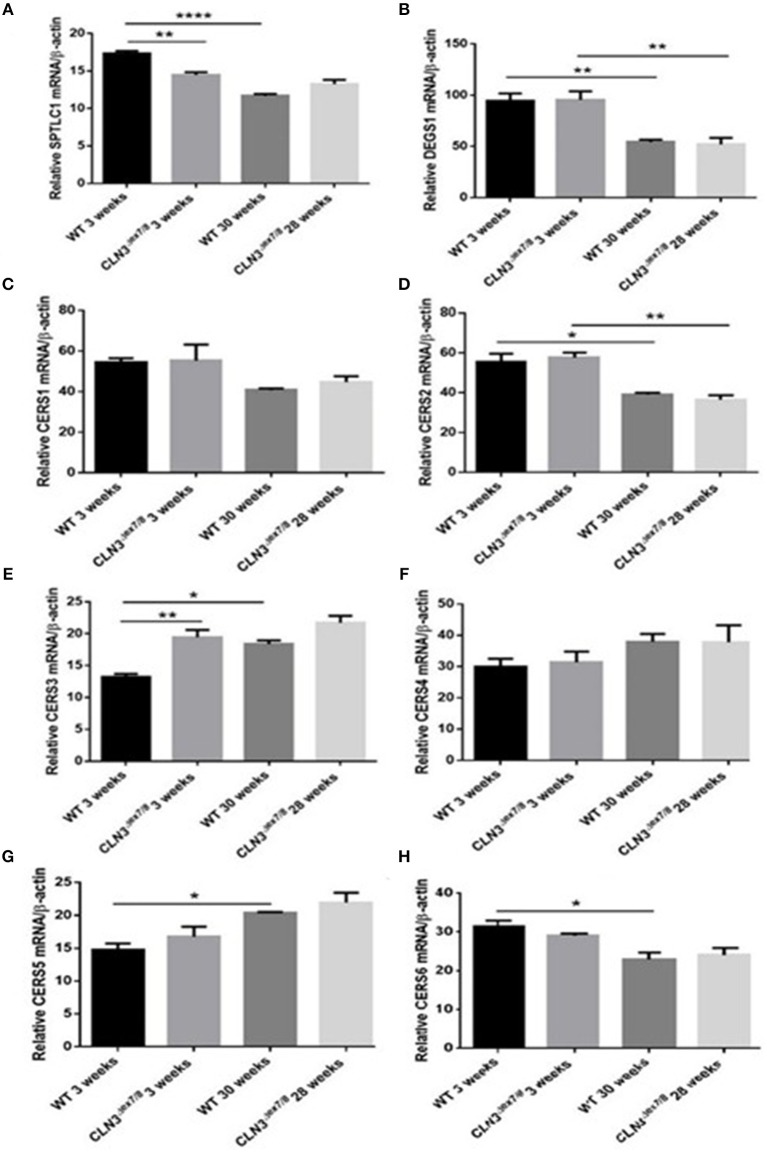
Relative mRNA expression of *de novo* ceramide synthesis enzymes SPTLC1 **(A)** (^**^P < 0.01, ^****^P < 0.0001); DEGS1 **(B)** (^**^P < 0.01); CerS1 **(C)**; CerS2 **(D)** (^*^P < 0.05, ^**^P < 0.01); CerS3 **(E)** (^*^P < 0.05, ^**^P < 0.01); CerS4 **(F)**; CerS5 **(G)** (^*^P < 0.05); CerS6 **(H)** (^*^P < 0.05) in WT and *Cln3*^Δ*ex7/8*^ mouse brain at 3 and 28–30 weeks of age (*n* = 3 in each group) by qRT-PCR. SPTLC1, DEGS1, CerS1, 2, and 6 are substantially upregulated in WT mouse brain at 3 weeks compared to 30 weeks of age. SPTLC1, CerS1, 3–6 show no significant change in expression between 3 and 28 weeks of age in *Cln3*^Δ*ex*7/8^ mouse brain. Only DEGS1 and CerS2 expression were significantly greater at 3 weeks compared to 28 weeks of age in *Cln3*^Δ*ex*7/8^ mice. The expression of *de novo* enzymes in *Cln3*^Δ*ex*7/8^ mouse brain at week 3 differed significantly from that in WT mice in only two of the enzymes: SPTLC1 shows less expression in *Cln3*^Δ*ex7/8*^ mice compared to WT mice, and CerS3 is overexpressed in *Cln3*^Δ*ex*7/8^ compared to age-matched WT mouse brain. At 28–30 weeks of age, differences in expression of *de novo* ceramide pathway enzymes in WT and *Cln3*^Δ*ex*7/8^ mouse brain were not statistically significant.

WT and *Cln3*^Δ*ex7/8*^ mouse mRNA levels of *de novo* ceramide synthesis enzymes SPTLC1, DEGS1, and ceramide synthases 1–6 (CerS1–6) were determined using qRT-PCR (Figure 2A). Enzymes involved in *de novo* synthesis of ceramide (SPTLC1, DEGS1, and CerS1, 2, and 6) are substantially upregulated in WT mouse brain at 3 weeks compared to 30 weeks of age (Figures 4A–D,H). This correlates well with the ceramide peak established in sera and brains of WT mice at 3 weeks. In *Cln3*^Δ*ex7/8*^ mice, the ceramide peak at 24 weeks in brain did not correlate with an increase in expression of *de novo* enzymes at week 28 compared to week 3 in brain of *Cln3*^Δ*ex7/8*^ mice. *De novo* enzymes (SPTLC1, CerS1, 3–6) (Figures 4A,C,E–H) show no significant change in expression between 3 and 28 weeks of age in brain, except for DEGS1 (Figure 4B) and CerS2 (Figure 4D) expression levels that were significantly greater at 3 weeks compared to 28 weeks of age in *Cln3*^Δ*ex7/8*^ mice. The expression of *de novo* enzymes in *Cln3*^Δ*ex7/8*^ mouse brain at week 3 differed significantly from that in age-matched WT mice in only two of the enzymes. SPTLC1, the rate-limiting step in ceramide synthesis, shows less expression in *Cln3*^Δ*ex7/8*^ mice compared to age-matched WT mice. CerS3 is overexpressed in *Cln3*^Δ*ex7/8*^ mouse brain compared to age-matched WT mice. At 28–30 weeks of age, differences in expression of *de novo* ceramide pathway enzymes in WT and *Cln3*^Δ*ex7/8*^ mouse brain were not statistically significant.

### Expression Analysis of Degradative Enzymes in the Ceramide Pathway

**Figure 5:**
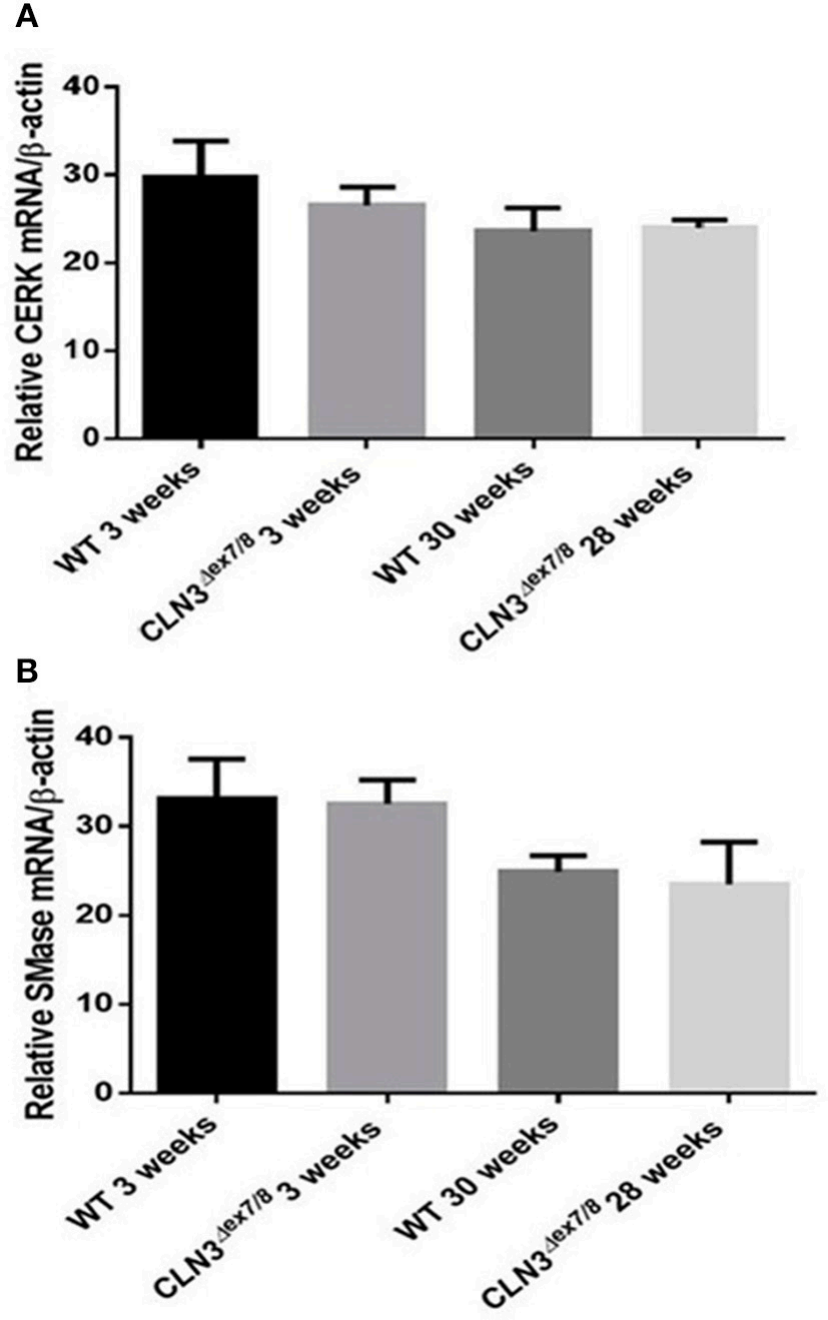
CerK **(A)** and SMase **(B)** relative mRNA expressions were not significantly different between WT and *Cln3*^Δ*ex7/8*^ mouse brain at 3 and 28–30 weeks (*n* = 3 in each group) as determined by qRT-PCR.

Ceramide can also be generated from hydrolysis of sphingolipids through the action of catabolic enzymes including acid sphingomyelinase (ASMase), a predominantly lysosomal protein, and neutral sphingomyelinases (NSMases) that localize to ER, Golgi, or cell membranes. Both ASMase and NSMases break down sphingomyelin to produce ceramide and phosphocholine (15, 17, 28). NSMase 2, the best characterized SMase, localizes to the cell membrane (15) and has been shown to function as a growth suppressor in mammalian cell lines (29). NSMase 2 expression using qRT-PCR does not show significant differences between WT and *Cln3*^Δ*ex7/8*^ mice at 3 and 28–30 weeks (Figure 5B). This suggests that NSMase 2 does not participate in the generation of the normal ceramide peak observed at 3 weeks in WT mice or in the dysregulation of ceramide levels in *Cln3*^Δ*ex7/8*^ mouse brain. Another important enzyme in ceramide metabolism is ceramide kinase (CerK). This enzyme phosphorylates ceramide but does not participate in its production. It is still essential to establish CerK mRNA expression levels in brain tissues, given that mouse brain exhibits high CerK activity (30). CerK mRNA expression showed no significant differences between normal and affected mouse brain at the different time points, so its activity was not altered coincident to the ceramide peak in WT or *Cln3*^Δ*ex7/8*^ mouse brain (Figure 5A).

### mRNA Expression Analysis of Pre-synaptic and Post-synaptic Proteins

**Figure 6:**
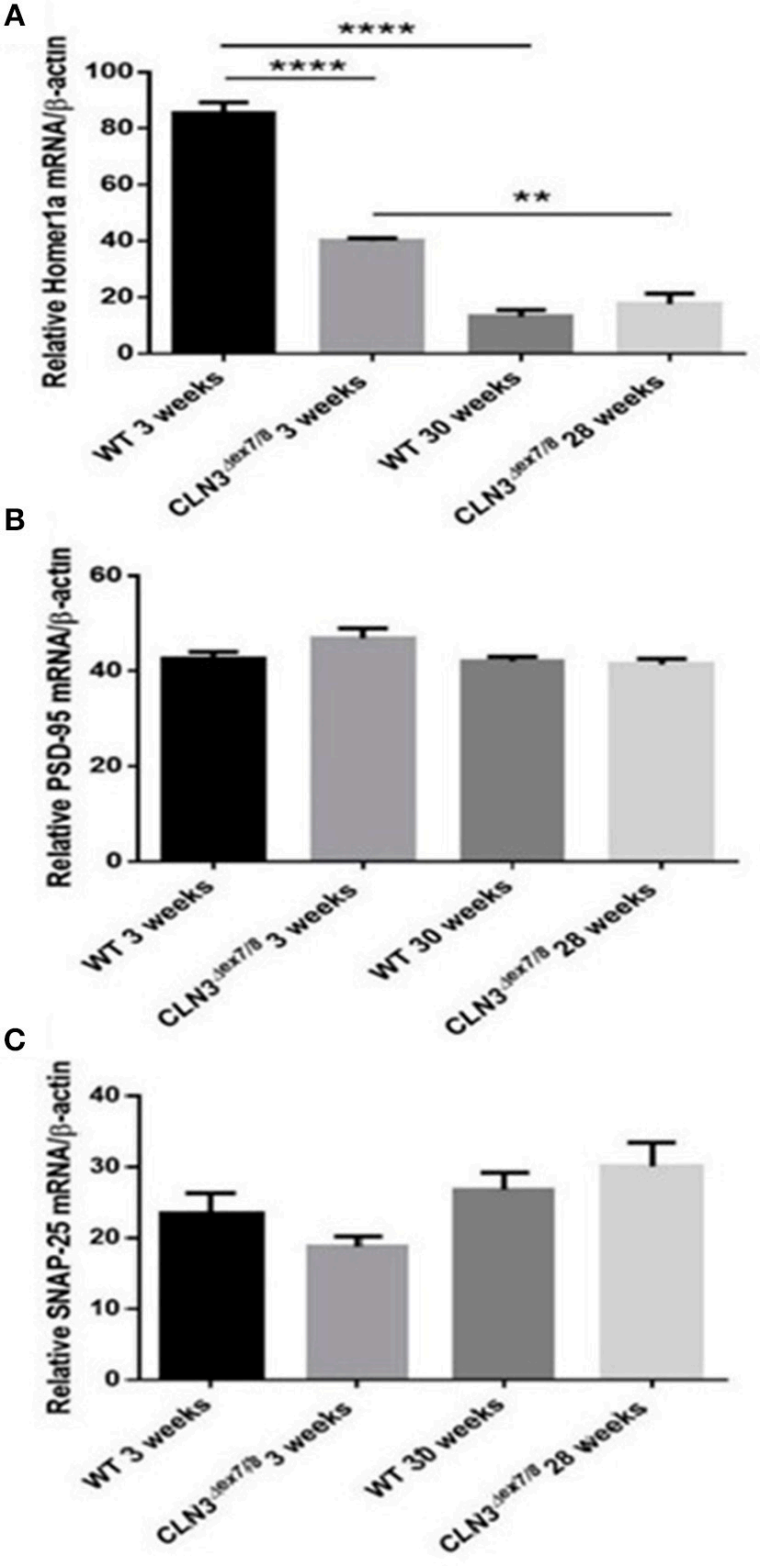
Relative mRNA expression of post-synaptic protein Homer-1a **(A)** (^**^P < 0.01 and ^****^P < 0.0001), post-synaptic protein PSD-95 **(B)** and pre-synaptic protein SNAP-25 **(C)** in WT and *Cln3*^Δ*ex7/8*^ mouse brain at 3 and 28–30 weeks of age (*n* = 3 in each group) as determined by qRT-PCR.

Synaptogenesis is critical during the first three postnatal weeks of life in rodents (31). Pre-synaptic protein SNAP-25 and post-synaptic proteins Homer-1a and PSD-95 mRNA levels were measured in WT and *Cln3*^Δ*ex7/8*^ mouse brain at 3 and 28–30 weeks of age. SNAP-25 tends to be higher at older ages in both WT and *Cln3*^Δ*ex7/8*^ mice, but differences were not statistically significant (Figure 6C). PSD-95 remains unchanged between normal and affected mice at the different time points (Figure 6B). Homer-1a, which is upregulated by synaptic activity as an immediate early gene, is overexpressed in WT and *Cln3*^Δ*ex7/8*^ mouse brain at 3 weeks of age compared to 30 weeks-old mice (Figure 6A). At 3 weeks of age, however, Homer-1a expression was significantly lower in *Cln3*^Δ*ex7/8*^ mouse brain compared to age-matched WT mice.

### PARP Cleavage and mRNA Expression Analysis of Caspases 3/6/9 and Cytochrome C As a Hallmark for Apoptosis

**Figure 7:**
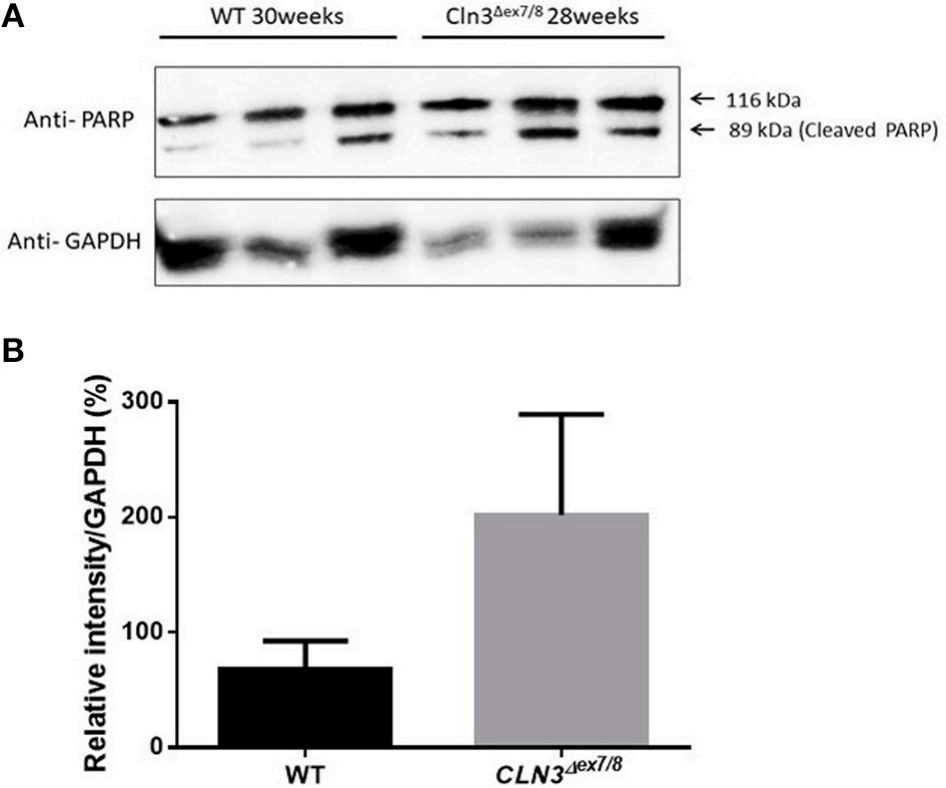
Quantification of apoptosis as determined with PARP cleavage by Western blot of WT and *Cln3*^Δ*ex7/8*^ mouse brain (*n* = 3 in each group). **(A)** Representative image of Western blot analysis. **(B)** Mean relative intensity quantification of three independent experiments. There is an increase in apoptosis in 28–30 weeks of age *Cln3*^Δ*ex7/8*^ compared to age-matched WT mouse brain.

**Figure 8:**
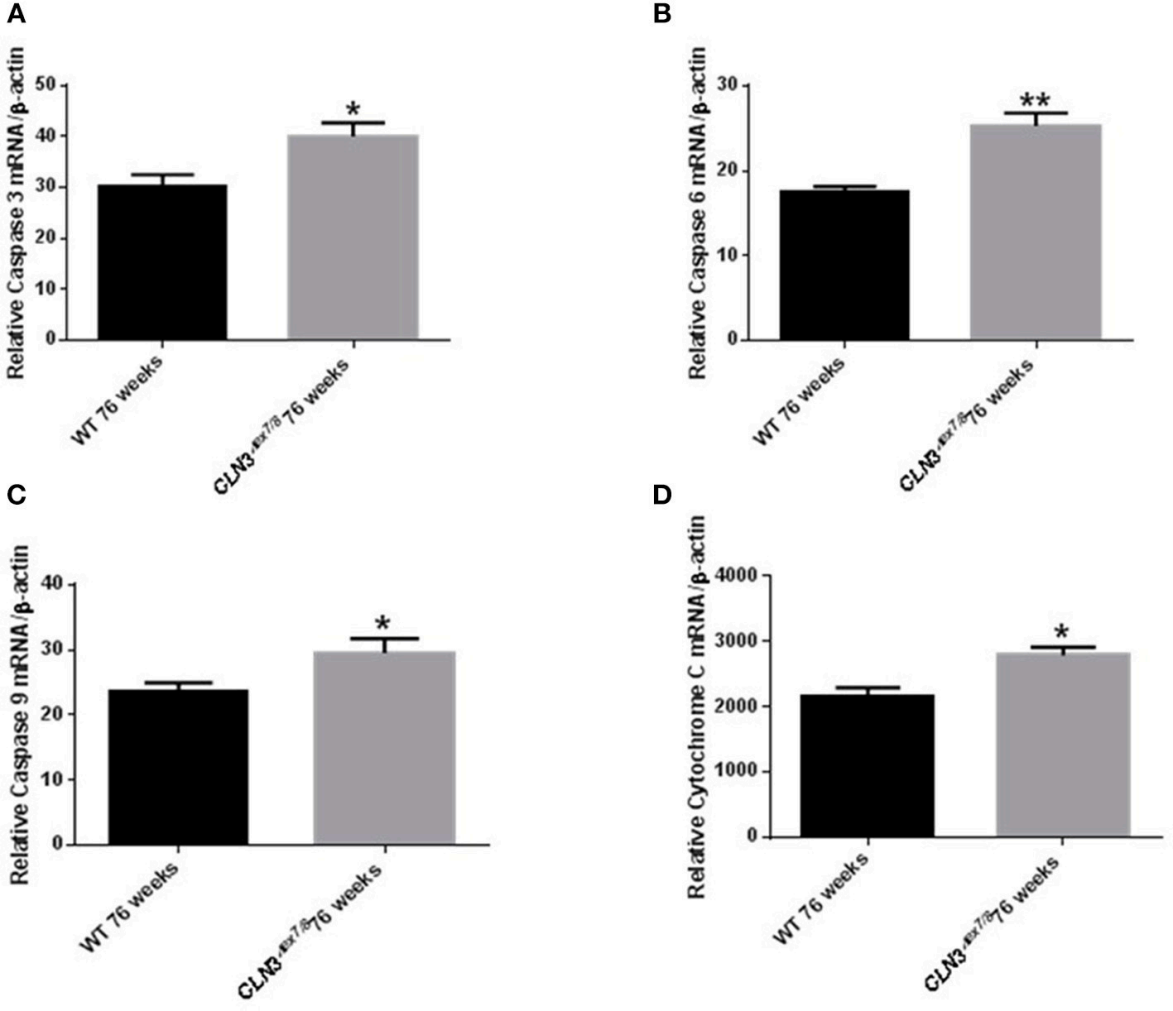
Relative mRNA expression of apoptosis markers Caspases 3 **(A)**, 6 **(B)**, 9 **(C)**, and cytochrome C **(D)** in WT and *Cln3*^Δ*ex7/8*^ mouse brain at 76 weeks of age (*n* = 4 in each group) by qRT-PCR. Caspases 3 (^*^P < 0.05), 6 (^**^P < 0.01), 9 (^*^P < 0.05) and cytochrome C (^*^P < 0.05) are substantially upregulated in *Cln3*^Δ*ex7/8*^ mouse brains compared to age-matched WT mice at 76 weeks of age.

Ceramide accumulates in the brains of CLN3 patients (6) and CLN3-deficient cells and is implicated in the pathogenesis of neuronal cell death (21). A pathological ceramide peak in brains of *Cln3*^Δ*ex7/8*^ mice was determined at 24 weeks (Figure 3D). At 28 weeks of age, there is increased PARP cleavage by Western blot analysis in *Cln3*^Δ*ex7/8*^ mouse brain compared to age-matched WT mouse brain (Figures 7A,B). The increase in apoptosis in brains of *Cln3*^Δ*ex7/8*^ mice trended toward significance, and followed the pathological ceramide peak in brains of *Cln3*^Δ*ex7/8*^ mice (Figure 3D). To confirm the results obtained by Western blot analysis, we investigated the mRNA expression of caspases 3, 6, 9, and cytochrome C in *Cln3*^Δ*ex7/8*^ mouse brains compared to age-matched WT mouse brains at 76 weeks of age. Caspases 3, 6, 9, and cytochrome C are significantly upregulated in *Cln3*^Δ*ex7/8*^ mouse brains compared to WT mice (Figures 8A–D).

## Discussion

This study describes for the first time ceramide levels in *Cln3*^Δ*ex7/8*^ mouse brains and sera, and establishes temporal changes in ceramide levels in normal vs. *Cln3*^Δ*ex7/8*^ mouse brains and sera. The use of serum ceramide levels as a biological marker has been established for other neurological (32, 33) and non-neurological diseases such as chronic kidney disease and Alzheimer's disease (34, 35), but not in CLN3 disease. Molecular DNA confirmation of CLN3 disease remains the diagnostic method of choice. Ceramide dysregulation, however, is implicated in the pathogenesis of CLN3 disease (6, 9, 21) and is increased in CLN3-deficient cells and CLN3 disease patient brains (6) and sera (El-Sitt et al., p. 74, NCL 2018 London Programme Abstract). Conversely, ceramide levels are decreased in *CLN3*-overexpressing cells (10). Elevated ceramide levels in the sera of CLN3 disease patients probably mirrors ceramide overproduction in patient brains. This paves the way for more research on human subjects that should aim to establish ceramide serum levels as a potential patient accessible methodology for tracking response to novel therapeutic strategies in CLN3 disease.

*Cln3*^Δ*ex7/8*^ mouse sera exhibit significantly higher levels of ceramide compared to WT mice. This strongly supports the ceramide serum data previously established from CLN3 patients and suggests again the potential use of serum ceramide levels as a biological tracker for CLN3 disease in humans and in the *Cln3*^Δ*ex7/8*^ mouse model for evaluation of emerging therapies in affected mice and humans. Tracking the therapeutic response of lowered serum ceramide levels in *Cln3*^Δ*ex7/8*^ mice in response to exogenous galactosylceramide is also documented (El-Sitt et al., p. 74, NCL 2018 London Programme Abstract).

*Cln3*^Δ*ex7/8*^ mouse brains also exhibit higher levels of ceramide compared to WT mice. LC-MS analysis uncovered, more specifically, that Cer16-, Cer18-, Cer18:1-, Cer22-, and Cer22:1-ceramides were higher in *Cln3*^Δ*ex7/8*^ mouse brains compared to WT mice, with the highest being Cer18-ceramide, previously described to be the primary fatty acyl Co-A in mouse brain (36). Differences in ceramide fatty acid chain length levels are probably due to variable expression levels and activity of distinctive ceramide synthases (CerS1–6) in WT and *Cln3*^Δ*ex*7/8^ mouse brains. Whole mouse brain expresses mainly three ceramide synthases (CerS1, 2, and 6) (27). CerSs are a group of enzymes that catalyze the formation of ceramide from a sphingoid base and specific acyl-CoA substrates, as part of the *de novo* sphingolipid synthesis pathway (37). Enzymes involved in *de novo* synthesis of ceramide (DEGS1, CerS1, 2, 4, 5, and 6) are substantially upregulated in *Cln3*^Δ*ex7/8*^ whole mouse brains compared to age-matched WT mice. This supports the increase of total ceramide levels in *Cln3*^Δ*ex7/8*^ compared to WT mouse brain.

The pattern of ceramide levels in serum and brain of WT mice at different agesis valuable baseline information on developmental brain and serum ceramide in normal mice and is useful for studying sphingolipid metabolism in the *Cln3*^Δ*ex7/8*^ mouse model and other disease mouse models with abnormalities in sphingolipid content. In WT mice, serum and brain ceramide levels correlate well, both significantly peaking at 3 weeks of age, while remaining relatively low and unchanged from ages 3 to 48 weeks. This pattern represents ceramide developmental regulation in the normal mouse and is pivotal for the study of ceramide dysregulation in diseases such as CLN3 disease. The concordance between serum and brain ceramide levels in normal mice is an important prerequisite for use of serum ceramide as a disease marker for monitoring emerging therapies for CLN3 disease in *Cln3*^Δ*ex7/8*^ mice. In parallel to the ceramide peak at week 3, there is upregulation in expression of the following ceramide synthesis enzymes in normal mouse brain: SPTLC1, DEGS1, and CerS1, 2, and 6. There was, however, no increase in expression of CERK or SMase. The ceramide peak in WT mice is primarily driven by upregulation of *de novo* ceramide synthesis enzymes, and not from sphingomyelin breakdown. This peak suggests a developmental role for ceramide at this age.

The critical period of synaptogenesis in rodents occurs during the first 3 weeks of life (31). Also, ceramide has been implicated in dendritic differentiation and survival of cerebellar purkinje cells (38) and in survival and maturation of immature hippocampal neurons (39, 40). The Homer-1a gene codes for a key post-synaptic protein and is significantly upregulated in WT mouse brain at 3 weeks of age, coincident with the serum ceramide peak suggesting upregulated synaptic activity (41). These results support a role for ceramide in mediating development of neuronal cells, dendritic differentiation, and synaptogenesis at the age of 3 weeks in normal mice.

CerS2 is upregulated in the brains of 3 weeks-old mice and is involved in synthesis of ceramides with long chain (C22–24) fatty acid residues (42). These ceramide species are implicated in synthesis of myelin sphingolipids, suggesting that CerS2 may be a limiting factor in ceramide synthesis during myelination (42). CerS2-deficient mice demonstrate progressive loss of myelin and compacted myelin and myelin basic protein (MBP) by 50 and 80%, respectively (43). In WT and *Cln3*^Δ*ex7/8*^ mice, CerS2 is upregulated at week 3 relative to 28–30 weeks of age, suggesting that this physiological peak in myelination at 3 weeks of age in normal mice is intact in *Cln3*^Δ*ex7/8*^ mice.

Establishing normative ceramide levels in WT mice is crucial for achieving a better understanding of the ceramide pathway in the *Cln3*^Δ*ex7/8*^ mouse model and other disease mouse models. Compared to WT mice, *Cln3*^Δ*ex7/8*^ mice display a disturbance in regulation of brain ceramide levels and synaptic protein mRNA expression. In serum, there is an early ceramide peak at 1 week and not at 3 weeks of age. The ceramide peak in *Cln3*^Δ*ex7/8*^ mouse brain appears at 24 weeks of age and does not correlate with serum levels. Absence of a ceramide peak at 3 weeks of age in *Cln3*^Δ*ex7/8*^ mouse brain may imply that the physiological increase in ceramide is negatively impacted by the absence of CLN3 protein through an unknown mechanism, or that brain ceramide levels are maintained higher than normal throughout the lifespan of *Cln3*^Δ*ex7/8*^ mice, obscuring the physiological peak at 3 weeks of age. All possibilities point to dysregulation in ceramide levels that interfere with normal brain development. SPTLC1 activity, a rate-limiting occurrence in ceramide *de novo* synthesis, is downregulated in *Cln3*^Δ*ex7/8*^ mouse brain at 3 weeks of age, hinting at the absence of the ceramide peak at 3 weeks. Expression of CerS3 in *Cln3*^Δ*ex7/8*^ mouse brain is upregulated relative to WT mouse brain at 3 weeks of age. This difference in expression might be insignificant, given the fact that CerS3 has almost undetectable expression in mouse brain (42) and shows high levels in testes and skin (44, 45). Alternatively, it may be an attempt to compensate for the absence of the ceramide peak at 3 weeks of age.

In *Cln3*^Δ*ex7/8*^ mice, early disturbance in synaptic activity is suggested by the significant downregulation of Homer-1a in the brain at 3 weeks of age and raises the possibility that an early insult to the brains of CLN3 patients may be responsible in part for later clinical manifestations. Additionally, dysregulated serum ceramide levels may be potentially used to mirror dysregulated brain ceramide levels and disease activity.

The non-physiological peak in brain ceramide levels in *Cln3*^Δ*ex7/8*^ mice at 24 weeks of age, equivalent to ~20–30 years of age in human adults (46), reflects a pathological increase in ceramide that may explain increased neuronal cell death and perhaps contributes to the demise of CLN3 disease patients during the third decade of life.

The increase in brain ceramide levels does not correlate with an increase in expression of *de novo* ceramide synthesis enzymes at 28 weeks. Activation of the PARP-cleaving caspase observed at 28 weeks of age in affected *Cln3*^Δ*ex7/8*^ mice, however, correlates with a peak in brain ceramide levels. This is not reflected in concomitant mRNA regulation of *de novo* ceramide synthesis enzymes and may be explained by covalent modification, allosteric regulation (22) or other modifying factors, such as decreased degradation of the actual *de novo* ceramide synthesis enzyme proteins. The increase in *de novo* ceramide synthesis enzymes was confirmed at 44 weeks of age when there is correlation with even more increased apoptosis at that time.

Initiation of apoptosis by caspases is well-documented in CLN3 patient cells (21). Inhibition of BCL-2 expression, release of cytochrome C, and activation of caspases 3/6/9 result in an apoptotic cascade. Activation of the PARP-cleaving caspase observed at 28 weeks of age in affected *Cln3*^Δ*ex7/8*^ mice, was reflected in mRNA regulation of apoptotic markers studied (Caspases 3, 6, 9, and cytochrome C) in affected *Cln3*^Δ*ex7/8*^ mice at 76 weeks of age. This age discrepancy may be explained by the fact that at 28–30 weeks of age, it is still early to detect apoptosis, so by looking at a later time point in mice (76 weeks), where there is even more increased apoptosis, we found significantly elevated cytochrome C and Caspases 3/6/9 mRNA expression in *Cln3*^Δ*ex7/8*^ mouse brains compared to WT mice.

Ceramide accumulates in brains of CLN3 disease patients (6). In patient-derived cells, ceramide accumulation correlates with activation of the apoptosis pathway upstream of initiator caspases 8 and 9 (21), leading to neuronal death. This is the first study describing temporal changes in ceramide levels and apoptosis over the lifespan of WT and *Cln3*^Δ*ex7/8*^ mice. A ceramide peak in the brain of the *Cln3*^Δ*ex7/8*^ mouse model at 24 weeks of age precedes neuronal apoptosis in *Cln3*^Δ*ex7/8*^ mouse brain observed at 28 and 76 weeks of age.

This study describes for the first time developmental changes in ceramide levels in normal and *Cln3*^Δ*ex7/8*^ mouse brains and sera in addition to apoptosis in normal and *Cln3*^Δ*ex7/8*^ mouse brains. In normal mouse sera and brains, a ceramide peak at 3 weeks of age may mediate developmental neuronal cell survival, dendritic differentiation, and synaptogenesis, and the absence of a similar peak in *Cln3*^Δ*ex7/8*^ mouse might be related to early disease pathogenesis. On the other hand, upregulation of ceramide in *Cln3*^Δ*ex7/8*^ mouse brains at 24 weeks of age precedes documented neuronal apoptosis. In human subjects, the previously described elevated ceramide levels in sera of CLN3 disease patients paves the way for establishing patient lowered serum ceramide levels as a measure for determining therapeutic effects of emerging therapies for CLN3 disease, which has already been shown in *Cln3*^Δ*ex7/8*^ mice in response to therapeutic strategies (El-Sitt et al., p. 74, NCL 2018 London Programme Abstract).

## Author Contributions

SE-S, JS, JA, and JM performed experiments, analyzed data, interpreted the results, prepared the figures, and drafted the main manuscript. NM and HH participated in performing experiments. R-MB conceived the study, obtained funding for the study, designed experiments, reviewed data and analyses, and revised and edited the manuscript. All authors reviewed and approved the final manuscript.

### Conflict of Interest Statement

The authors declare that the research was conducted in the absence of any commercial or financial relationships that could be construed as a potential conflict of interest.
