# Supplementary material for: Developmental Comparison of Ceramide in Wild-Type and Cln3Δex7/8 Mouse Brains and Sera
Source: Front Neurol. 2019 Feb 19;10:128. doi: 10.3389/fneur.2019.00128 (PMC6389635; doi:10.3389/fneur.2019.00128)
Supplement: Supplementary file 1 [file Presentation_1.PPTX]

## Slide 1
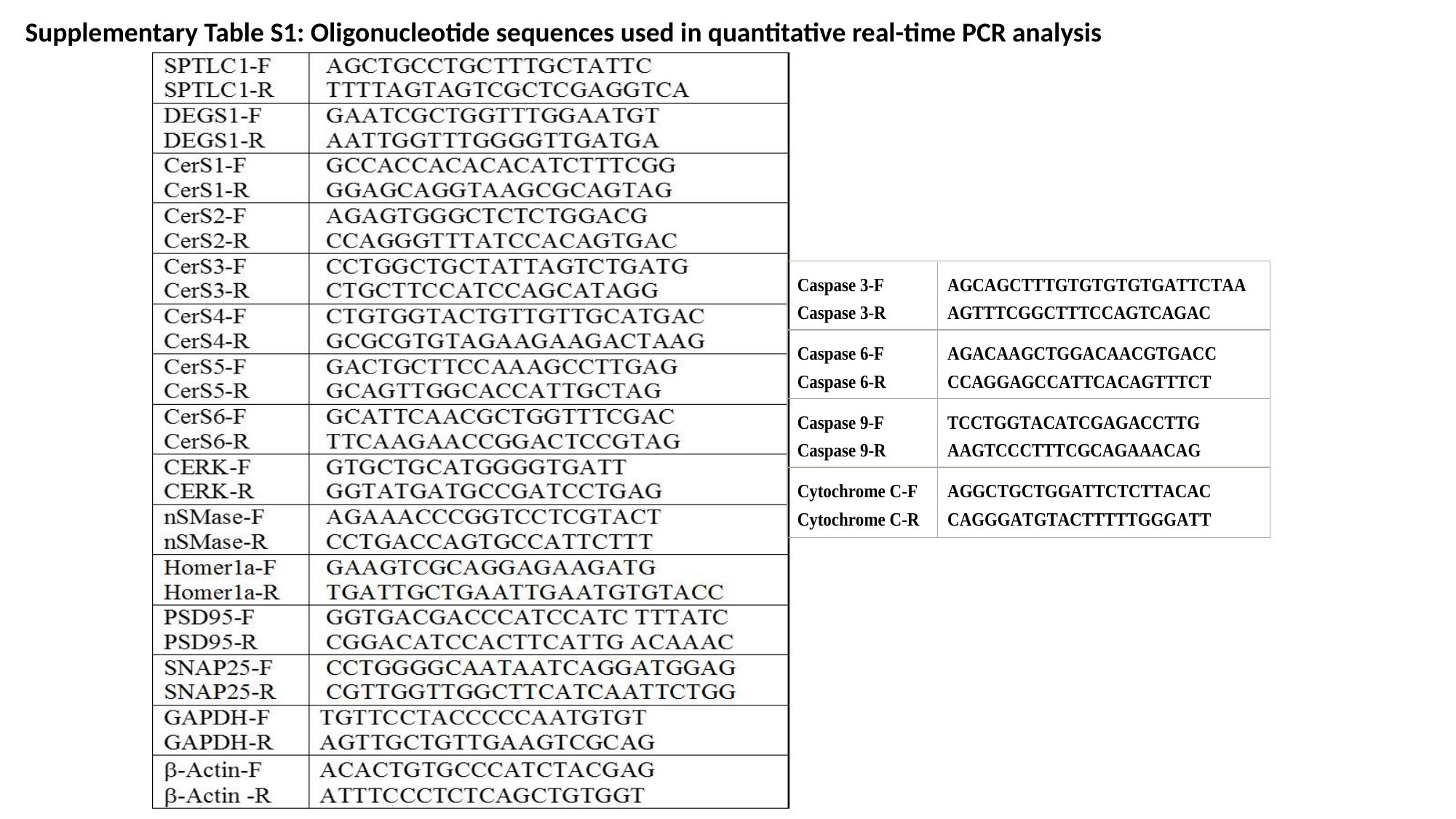

Supplementary Table S1: Oligonucleotide sequences used in quantitative real-time PCR analysis
